# Clinical profile of carpal tunnel syndrome in a teaching hospital

**DOI:** 10.12669/pjms.291.2946

**Published:** 2013

**Authors:** Hussein Mohammed Malibary, Afnan Tawfeeg Al-Najjar, Dina Mohammed Yassen, Hamad Abdullah Almarzouki Abuhussain, Osman Omer Radhwi, Zainab Ridha Alfares

**Affiliations:** 1 Hussein Mohammed Malibary, MBBS, PhD, Department of Neuromedicine, King Abdulaziz University Hospital, Jeddah, Saudi Arabia.; 2 Afnan Tawfeeg Al-Najjar, MBBS, Department of Internal Medicine, King Fahd Medical City, Riyadh, Saudi Arabia.; 3 Dina Mohammed Yassen, MBBS, Department of Internal Medicine, King Abdulaziz Medical City, Jeddah, Saudi Arabia.; 4 Hamad Abdullah Almarzouki Abuhussain, MBBS, Department of Internal Medicine, King Abdullah Medical City, Makkah, Saudi Arabia.; 5 Osman Omer Radhwi, MBBS, Department of Hematology/Oncology, King Abdulaziz University Hospital, Jeddah, Saudi Arabia.; 6 Zainab Ridha Alfares, MBBS, King Abdulaziz University Hospital, Jeddah, Saudi Arabia.

**Keywords:** Carpel tunnel syndrome, Surgical decompression, Nerve conduction

## Abstract

***Objectives: ***The aim of this study was to review the clinical characteristics and demography of Carpel Tunnel Syndrome (CTS) cases presented to a university hospital.

***Methodology: ***A retrospective study was done for 336 consecutive patients (290 females and 46 males), referred with a clinical diagnosis of CTS to the Neuro-diagnostic laboratory at King Abdulaziz University Hospital, Jeddah, Saudi Arabia, between January 2007 and December 2010. All subjects had clinical evaluation and standardized nerve conduction studies (672 hands) performed by the same examiner.

***Results: ***Carpal tunnel syndrome was confirmed in 640 hands (95.23%) with female predominance (86.3%). The mean age was 52.4 in females and 54.4 years in males with overall highest occurrence among the age group 45- < 55 years. Bilateral CTS was confirmed in the majority of the patients, i.e., 304(90.5%), and remaining were unilateral. Among unilateral pattern, 22 (68.8%) had right CTS and others had left CTS. Most of the patients were treated conservatively 85.4% while the rest had surgical decompression 14.6%.

***Conclusion: ***Overall predominant age group was 45-<55 years with female dominancy. Majority of subjects had bilateral CTS as well as conservative treatment was frequent.

## Introduction

 Carpal Tunnel Syndrome (CTS) is the most common compressive peripheral neuropathy in the upper extremity. Its classic symptoms are numbness and paraesthesia in the first three fingers of the hand, which is commonly exacerbated at night. The diagnostic signs include sensory loss along the lateral aspect of the hand, motor weakness and wasting of *Abductor Pollicis Brevis* (APB) muscle, and eliciting Tinel’s and Phalen’s sign at the wrist. The nerve conduction study (NCS) study is a definite diagnostic test for CTS.^1^^,^^2^

 The estimated prevalence of CTS in adults in the United Kingdom is 7 to 16%, and the number of elective hand surgeries for CTS has almost doubled over the decade from 1990 to 2000. CTS patients surgically treated in the United States number between 400,000 and 500,000 per year and the associated annual economic cost is in excess of $2 billion.^3^^-^^6^ Congenital predisposition is the commonest cause of CTS in which carpal tunnel is simply narrower in some people than in others especially middle aged people. Other factors which contribute in its causation are stressful work, injury, trauma, endocrine disorders, joint deformities, fluid retention, and the development of any space occupying lesions in the tunnel.^7^^-^^9^

 The aim of this study was to review the clinical characteristics and demography of CTS cases presented to a university hospital.

## Methodology

 The retrospective study was based upon patients’ files review that were referred with a suspected diagnosis of CTS over a period of 48 months from January 2007 to December 2010 at the department of neurosciences of King Abdul Aziz University Hospital, Jeddah, Saudi Arabia.

 The files were reviewed for clinico-epidermiological parameters which included: age, gender (Male and female), symptomatic side (left and/ right), neurophysiological grades, i.e., mild, moderate, severe and very severe according to American Association of Electrodiagnostic Medication (AAEM) and predisposing factors. The patients were divided into different age groups (Table-I). Neurophysiological grades were defined as (a) mild CTS: prolonged distal sensory peak latency with ± decreased sensory amplitude, (b) moderate CTS: abnormal median sensory peak latencies with prolongation of the distal motor latency, (c) severe CTS: prolonged motor and sensory distal peak latency either with a low or absent sensory nerve action potential (SNAP) or compound muscle action potential (CMAP), (d) very severe CTS: absent thenar motor or sensory response either with a present or absent lumbrical response.

 Motor and sensory nerve conduction study (NCS) of median nerve was performed in both hands, unless the patient refused. The temperature was maintained at >32ºC during the procedure. For motor NCS, the median motor nerve was stimulated at wrist 6.5 cm proximal to the active recording electrode. The sensory responses were obtained at digit II and digit V for the median nerve, stimulating antidromically at 13 cm and 11 cm, respectively. The normative value in our laboratory for median motor latency is <4.5 milliseconds and median sensory distal peak latency <3.5 milliseconds.

 The difference in sensory peak latency of ≥ 0.4 milliseconds was considered significant. The latency difference was measured at digit IV for the median sensory nerve was measured at the wrist at a distance of 14 cm, and a difference in peak latency by ≥ 0.4 ms was considered significant. In the patients with bilateral CTS, the neurophysiological grade in the more severely affected hand was noted.

 Data was analyzed by using Microsoft Excel version 7 on personal computer and subjected to descriptive analysis. Categorical data was analyzed as number (percentage).

***Ethical Issues: ***The institutional review board approval was taken from hospital board of directors after they had been made aware of purpose of study. We declare that we have no financial or personal relationship(s) which may have inappropriately influenced us in writing this paper.

## Results

 A total of 336 patients with 640 hands including 290 females with a mean age of 52.4 years and 46 males with a mean age of 54.4 years were found in the group studied. The female to male ratio was 6.3:1. The overall peak of CTS was found in age group of 45-<55 years. Table-I.

 Co-existing diseases were found as diabetes mellitus (DM) in 132 (35.9%), hypothyroidism in 52 (14.1%) and pregnancy in 2 (0.5%) patients while five (1.4%) patients were on dialysis and only one (0.3%) was taking isoniazid.

 Mild form of CTS was most frequent in both right and left sides, i.e., 165(50.3%) and 183(58.7%), respectively. Bilateral CTS pattern was most frequent among females as well as males, i.e., 296(92.8%) and 35(76.1%), respectively. Table-II.

 Majority of patients were treated conservatively 287 (85.4%), while remaining underwent surgical decompression. Of the patients who underwent surgical decompression, 27 (54.9%) had right hand surgery, 7 (41.2%) had left hand surgery and 2 (3.9%) had bilateral hand surgery.

## Discussion

 In our study the higher predominance of CTS is in females with a ratio of 6.3:1 which was comparable to other studies, i.e., 5.6:1, 5.4:1, 10:2, 5:1 and 4.9:1.^10^^-^^14^ Mean age of CTS subjects in our study was found to be 52.4 years in females and 54.3 years in males and the peak age was 45-<55 years. Abumunaser reported mean age 45.5 years in females and 48.5 in males and the peak age was 45-54 years which was similar to our study.^14^ Al-Sulaiman and Ismail reported the mean age in females was 37 years and 44 years in males and the peak age was 31-40 years which is younger than our study group.^13^ DM was the most common associated disease to CTS 35.9% followed by hypothyroidism 14.1%. These findings are in conformity with other studies in which DM was the higher risk factor.^10^^,^^11^^,^^14^ About 90.5% of our patients had bilateral CTS which is higher than other reported data while 9.5% of our patients had unilateral CTS.^10^^,^^11^^,^^14^ Majority of patients in our study had conservative treatment 85.4%, while 14.6% had surgical decompression. This result is similar to other reported studies. In the study of Bahou YG, 140 out of 185 had conservative treatment while the rest 45 patients had surgical decompression.^11^ In Tay LB et al., 88 of patients had conservative treatment while 27 patients had surgical decompression.^12^

**Table-I:** Demographical data (n=336).

*Gender*	*n*	*%*
Female	290	86.3
Male	46	13.7
Age Groups in years		
15-<25	5	1.5
25-<35	22	6.5
35-<45	60	17.9
45-<55	106	31.5
55-<65	79	23.5
65-<75	46	13.7
≥75	18	5.4

**Table-II:** CTS Categorization with Pattern among Gender.

*CTS categorization (n=640)*		*Right*	*Left*
	Mild	165(50.3)	183(58.7)
	Moderate	158(48.2)	85(27.2)
	Severe	1(0.3)	36(11.5)
	Very Severe	4(1.2)	8(2.6)
	Total	328(100)	312(100)
*CTS pattern among Gender (n=336)*		*Unilateral*	*Bilateral*
	Female	21(65.6)	269(88.5)
	Male	11(34.4)	35(11.5)
	Total	32(100)	304(100)

This study was confined to demographic profile with CTS category and pattern. It has the importance in the perspective of highlighting the consistency/inconsistency of our findings with those of previous Saudi Arabian and other Middle East researches to build a solid evidence of epidemiology, demographic and clinical profile of this disease. It emphasizes that health care planners and providers at government, private and personal level to initiate prevention and management plan in the light of local as well as international past and recent scientific research findings and updates. As notified in the introduction of current study about its causative factors, there is a need for development and implementation of causative factors prevention strategies like; obesity, diabetes mellitus, thyroid disorders, excessive unnecessary usage of hands, sedentary habits, and salt intake in fluid retention conditions. This can be possible by early detection and aggressive management of causative factors. However, causative factors like; sedentary habits, obesity, diabetes and unnecessary use of hand can also be prevented by effective healthy diet and lifestyle education and public awareness programmes on mass media. Future studies are required to find out correlating risk factors, associated diseases, in both genders.

## Conclusions

 In conclusion, the demographic pattern of CTS (age, site, and gender) in our study was almost similar to the pattern in other provinces of Saudi Arabia, and comparative to those reported in other studies in the developed countries. However, a slightly higher involvement of females was found than those of developed countries.
